# Surgical site infections following coronary artery bypass graft procedures: 10 years of surveillance data

**DOI:** 10.1186/1471-2334-14-318

**Published:** 2014-06-10

**Authors:** Damin Si, Mohana Rajmokan, Prabha Lakhan, John Marquess, Christopher Coulter, David Paterson

**Affiliations:** 1 Centre for Healthcare Related Infection Surveillance and Prevention, Queensland Health, Brisbane, Australia; 2 The Prince Charles Hospital, Brisbane, Australia; 3 University of Queensland Centre for Clinical Research, Brisbane, Australia

**Keywords:** Coronary artery bypass graft surgery, Surgical site infections, Pathogens, Risk factors

## Abstract

**Background:**

Surgical site infections following coronary artery bypass graft (CABG) procedures pose substantial burden on patients and healthcare systems. This study aims to describe the incidence of surgical site infections and causative pathogens following CABG surgery over the period 2003–2012, and to identify risk factors for complex sternal site infections.

**Methods:**

Routine computerised surveillance data were collected from three public hospitals in Queensland, Australia in which CABG surgery was performed between 2003 and 2012. Surgical site infection rates were calculated by types of infection (superficial/complex) and incision sites (sternal/harvest sites). Patient and procedural characteristics were evaluated as risk factors for complex sternal site infections using a logistic regression model.

**Results:**

There were 1,702 surgical site infections (518 at sternal sites and 1,184 at harvest sites) following 14,546 CABG procedures performed. Among 732 pathogens isolated, Methicillin-sensitive *Staphylococcus aureus* accounted for 28.3% of the isolates, *Pseudomonas aeruginosa* 18.3%, methicillin-resistant *Staphylococcus aureus* 14.6%, and *Enterobacter* species 6.7%. Proportions of Gram-negative bacteria elevated from 37.8% in 2003 to 61.8% in 2009, followed by a reduction to 42.4% in 2012. Crude rates of complex sternal site infections increased over the reporting period, ranging from 0.7% in 2004 to 2.6% in 2011. Two factors associated with increased risk of complex sternal site infections were identified: patients with an ASA (American Society of Anaesthesiologists) score of 4 or 5 (reference score of 3, OR 1.83, 95% CI 1.36-2.47) and absence of documentation of antibiotic prophylaxis (OR 2.03, 95% CI 1.12-3.69).

**Conclusions:**

Compared with previous studies, our data indicate the importance of Gram-negative organisms as causative agents for surgical site infections following CABG surgery. An increase in complex sternal site infection rates can be partially explained by the increasing proportion of patients with more severe underlying disease.

## Background

Surgical site infections (SSIs) following coronary artery bypass graft (CABG) procedures pose substantial burden on patients and healthcare systems, particularly from serious infections at sternal sites (e.g. deep incisional and organ/space SSIs). The total length of stay for patients with SSIs after CABG surgery is significantly longer than those without SSIs [1]. The estimated excess costs associated with deep sternal site infections were over $20,000 per patient in the late 1990s [2,3] and are likely to be substantially greater now.

There are known host (e.g. advanced age, obesity and diabetes) and procedural factors (e.g. wound class, duration of procedures and surgical technique) associated with increased risk of SSIs [4-8]. A number of effective infection control strategies can assist in reducing SSIs, including appropriate antibiotic prophylaxis, effective patient skin preparation, and surveillance of SSIs with feedback of appropriate data to surgeons and hospitals [4,9].

Many countries have implemented standardised surveillance systems to monitor and report SSIs after specific procedures, largely based on surveillance methods developed by the US Centres for Disease Control and Prevention (CDC) National Healthcare Safety Network (NHSN, formerly the National Nosocomial Infections Surveillance System, NNISS) [5]. In Australia, while there is no national level surveillance system in place, states and territories have instituted surveillance programs for monitoring healthcare-acquired infections (HAIs) [10]. Previous analysis of SSI surveillance data (2001–2005) from Queensland, Australia found the NNISS risk index was insufficient as a risk stratification tool for SSIs, and suggested investigation of risk factors for procedure-specific SSIs [11].

In this paper we describe the incidence of surgical site infections and causative pathogens following CABG procedures in three Australian hospitals over the period 2003–2012, and examine risk factors for complex sternal site infections.

## Methods

### Study population and surveillance data collection

The Centre for Healthcare Related Infection Surveillance and Prevention (CHRISP) initiated a standardised, computerised HAI surveillance system (the Electronic Infection Control Assessment Technology, eICAT) during 2001 in public hospitals in Queensland, Australia [12]. There were 166 public acute hospitals in Queensland, only three of which had capacity to perform CABG surgery. Surveillance data on SSIs following CABG procedures from these three hospitals have been provided to CHRISP since introduction of the system. The surveillance system did not cover private hospitals in Queensland. We analysed data collected from 2003 to 2012 to take advantage of a more robust data collection system following an initial two year implementation phase (2001–2002).

Active patient-based surveillance was undertaken by infection control practitioners in participating hospitals to identify eligible patients undergoing CABG procedures and follow up SSI cases. Patients meeting the following criteria were included: 1) aged 14 years or older; 2) undergoing a CABG procedure that was defined by International Statistical Classification of Diseases and Related Health Problems, 10th Revision, Australian Modification/Australian Classification of Health Interventions, 6th Edition (ICD-10-AM/ACHI, Block codes 672, 673, 674) [13]. The scope of CABG procedures was consistent with the CDC NHSN definition [14]; 3) survival ≥ 48 hours post procedure; and 4) the procedure was classified as clean or clean-contaminated. Post-discharge surgical site surveillance was conducted using a postal survey of patients 30 days after CABG procedures. Overall response rates for the postal survey ranged from 69% to 75% across hospitals, with relatively stable response rates over time. Cases of SSIs were identified based on the CDC definitions [15,16], which was endorsed by the Australian Commission on Safety and Quality in Healthcare [17]. Data quality assurance procedures were implemented routinely to ensure accuracy, completeness and consistency of data in the eICAT. Reports containing individual hospital and aggregated state data were provided to participating hospitals on a six-monthly basis, as part of strategies to improve infection control practice.

Ethical approval was granted from the Queensland Health Central Health and Medical Research Human Research and Ethics Committee.

### Microbiology

Microbiological testing was conducted by the three laboratories attached to the hospitals performing cardiac surgery. All three laboratories were managed by Pathology Queensland and shared common methods. Antimicrobial susceptibility testing conformed to the recommendations of the CLSI (Clinical and Laboratory Standards Institute) except after June 2012 when all laboratories adopted the EUCAST (The European Committee on Antimicrobial Susceptibility Testing) system of interpretative criteria.

### Surveillance data

Infection outcomes were categorised in terms of detection time, infection types, and incision sites. Infections were classified as in-hospital SSIs if occurring during the hospital stay, or post-discharge SSIs if detected after discharge and within 30 days post procedure (in case of implant in situ, the follow-up period was within one year). Infections also were classified as either superficial (involving skin/subcutaneous tissue) or complex (involving deep soft tissue, organ/space) infections [15]. Infections were recorded as sternal or harvest site infections.

Potential risk factors were submitted on the eICAT surveillance system and examined for their association with SSIs following CABG procedures: 1) patient characteristics including age, sex and American Society of Anaesthesiologists (ASA) score [18]. ASA score ranges from 1 to 5, indicating a patient being healthy (1), with mild systemic disease (2), with severe systemic disease (3), with severe systemic disease that is a constant threat to life (4), or a moribund patient who is not expected to survive without the operation (5); and 2) procedural factors such as priority of surgery (emergency vs. elective), types of CABG surgery, wound classification (clean vs. clean-contaminated), number of grafts, and use of antibiotic prophylaxis. Local infection control practitioners reviewed medical records and medication charts to determine whether prophylactic antibiotics were given for the operative procedure with the intent of preventing infection at the surgical site. Procedure duration documented in the eICAT system was excluded from use in our analysis, due to inconsistency of date formatting applied to the start time and end time of CABG surgery.

### Statistical analysis

Means, medians and proportions were used to summarise continuous, binary and categorical data as appropriate. The SSI rate was calculated as the number of infections per 100 procedures. 95% confidence intervals were provided to facilitate comparison of crude SSI rates.

A univariate logistic regression was performed to assess association of each of the potential risk factors with complex sternal site infections. Robust estimates of standard errors were employed to accommodate clustering of data within hospitals. Variables with *p* < 0.25 were considered as candidate risk factors to be included in a multivariate logistic regression model.

The multivariate logistic regression model was constructed using a backward selection approach with robust estimates of standard errors. Variables were eligible for removal at *p* ≥0.05. The Hosmer-Lemeshow test was employed to assess the goodness-of-fit for the model (*p* > 0.05 indicating good fit). Predictive performance of the model was evaluated by using a c-index derived from the area under the receiver operating characteristic (ROC) curve. The c-index ranges from 0.5 to 1.0, indicating predictive ability from no better than chance to perfect. The final model was internally validated based on 100 bootstrap samples [14]. All data analysis was performed using Stata (version 12.1, StataCorp, TX).

## Results

### Demographic and clinical characteristics of patients undergoing CABG surgery

Over the period 2003–2012, a total of 14,517 patients underwent CABG surgery (Table 1). Patients were predominantly male (78.4%), with a median age of 66 years. CABG surgery was mainly elective (89.8%) and performed with both sternal and graft site incisions (93.9%). The mean number of grafts was 2.9. The majority of patients were recorded as having an ASA score of 3 or 4, a clean wound, and antibiotic prophylaxis administered. Absence of antibiotic prophylaxis was documented in a minority of cases (3.3%), which was more likely to occur in emergent cases or in patients with severe underlying disease (indicated by an ASA score of 5).

**Table 1:** Demographic and clinical characteristics of patients undergoing coronary artery bypass graft (CABG) procedures, 2003-2012

Characteristic*	**N**	**%**
Number of hospitals	3	
Number of patients undergoing CABG procedures	14,517	
Sex		
Male	11,379	78.4
Female	3,138	21.6
Age, median (interquartile range), years	66 (58 – 73)	
Age category, years		
<55	2,357	16.2
55-64	3,941	27.2
65-74	5,150	35.5
75+	3,069	21.1
ASA (American Society of Anaesthesiologists) score		
1	6	0.1
2	164	1.1
3	7,787	54.1
4	6,349	44.1
5	83	0.6
Priority of surgery		
Emergency	1,462	10.2
Elective	12,896	89.8
Types of CABG		
CABG with both sternal and graft site incisions	13,637	93.9
CABG with sternal site incisions only	880	6.1
Wound classification		
Clean	14,286	98.4
Clean-contaminated	228	1.6
Mean number of grafts	2.9	
Category of graft numbers		
1	1,394	9.6
2	3,218	22.2
3	5,846	40.3
4	3,197	22.1
5+	837	5.8
Antibiotic prophylaxis		
Yes	13,851	96.7
No	466	3.3

*Five variables with missing values: ASA score (128, 0.9%), priority of surgery (159, 1.1%), wound classification (3, 0.02%), category of graft numbers (25, 0.2%), and antibiotic prophylaxis (200, 1.4%).

### SSIs following CABG procedures

Twenty-nine of the 14,517 patients had a repeat CABG surgery over the reporting period, resulting in a total of 14,546 sternal site incisions, and 15,781 harvest site incisions (Table 2).

**Table 2:** Surgical site infections following coronary artery bypass graft procedures, by infection type and detection time, 2003–2012

**Infection type/detection time**	**Sternal site incisions (N = 14,546)**^**#**^			**Harvest site incisions (N = 15,781)**		
	**n**	Rate* (95% CI)	**%**	**n**	Rate* (95% CI)	**%**
Superficial SSIs	331	2.3 (2.0-2.5)		1,144	7.3 (6.9-7.7)	
In-hospital	151	1.0 (0.9-1.2)	45.6	299	1.9 (1.7-2.1)	26.1
Post-discharge	180	1.2 (1.1-1.4)	54.4	845	5.4 (5.0-5.7)	73.9
Complex SSIs	187	1.3 (1.1-1.5)		40	0.3 (0.2-0.4)	
In-hospital	111	0.8 (0.6-0.9)	59.4	25	0.2 (0.1-0.2)	62.5
Post-discharge	76	0.5 (0.4-0.7)	40.6	15	0.1 (0.1-0.2)	37.5
All infections	518	3.6 (3.3-3.9)		1,184	7.5 (7.1-7.9)	
In-hospital	262	1.8 (1.6-2.0)	50.6	324	2.1 (1.8-2.3)	27.4
Post-discharge	256	1.8 (1.6-2.0)	49.4	860	5.5 (5.1-5.8)	72.6

*Per 100 procedures.

^#^The total number of sternal site incisions (14,546) were more than the total number of patients (14, 517), as 29 patients underwent a repeat CABG surgery over the reporting period.

Among 14,546 sternal site incisions, 518 infections (3.6%) were identified, with one half of the cases detected in-hospital and the other half post-discharge. Of those sternal site infections, 187 (36.1%) were classified as complex SSIs. The complex sternal site infection rate was 1.3%.

For 15,781 harvest site incisions, 1,184 infection cases (7.5%) were reported, most of which were detected following discharge from hospital (72.6%), and were classified as superficial (96.6%).

### Pathogens causing SSIs

**Figure 1:**
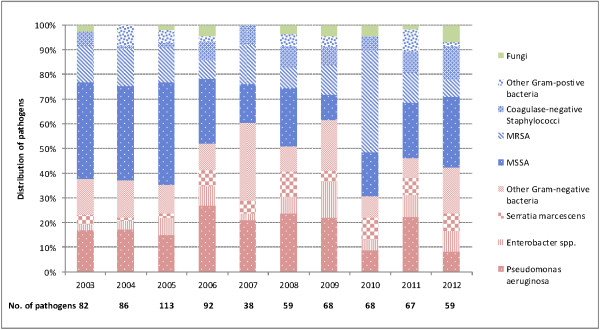
Distribution of pathogens causing surgical site infections following coronary artery bypass graft procedures, 2003–2012.

From 1,702 infections at either sternal or harvest sites, 732 cases (43.0%) had pathogenic organisms isolated (Table 3). Overall, Gram-positive bacteria were responsible for 53.0% of infections, Gram-negative bacteria 44.1%, and fungi (e.g. *Candida albicans)* 2.9%. Methicillin-sensitive *Staphylococcus aureus* (MSSA) were isolated from 28.3% of infection cases, followed by *Pseudomonas aeruginosa* (18.3%), methicillin-resistant *Staphylococcus aureus* (MRSA, 14.6%) and *Enterobacter* spp. (6.7%). Proportions of SSIs caused by Gram-negative organisms increased from 37.8% in 2003 to 52.2% in 2006 and 61.8% in 2009, followed by a reduction to 42.4% in 2012 (Figure 1).

**Table 3:** Pathogens causing surgical site infections following coronary artery bypass graft procedures, 2003-2012

**Pathogen**	**All surgical site infections**		**Complex sternal site infections**	
	**n**	**%**	**n**	**%**
Total number of infections	1,702		187	
Number of infections with pathogens isolated	732		152	
**Gram-positive - subtotal**	388	53.0	107	70.4
Methicillin-sensitive *Staphylococcus aureus*	207	28.3	50	32.9
Methicillin-resistant *Staphylococcus aureus*	107	14.6	20	13.2
Coagulase-negative *Staphylococci*	46	6.3	30	19.7
*Enterococcus* spp.	11	1.5	5	3.3
*Streptococcus* spp*.*	10	1.4	1	0.7
*Bacillus* spp*.*	3	0.4	-	-
*Peptostreptococcus* spp*.*	3	0.4	-	-
*Corynebacterium* spp*.*	1	0.1	1	0.7
**Gram-negative - subtotal**	323	44.1	41	27.0
*Pseudomonas aeruginosa*	134	18.3	5	3.3
*Enterobacter* spp*.*	49	6.7	9	5.9
*Serratia marcescens*	38	5.2	9	5.9
*Escherichia coli*	23	3.1	3	2.0
*Klebsiella* spp*.*	21	2.9	8	5.3
*Proteus mirabilis*	13	1.8	2	1.3
*Morganella morganii*	11	1.5	1	0.7
Other *Pseudomonas* spp*.*	14	1.9	-	-
*Citrobacter* spp*.*	8	1.1	2	1.3
*Acinetobacter baumannii*	5	0.7	1	0.7
Other Gram-negative bacteria*	7	1.0	1^#^	0.7
**Fungi - subtotal**	21	2.9	4	2.6
*Candida albicans*	20	2.7	4	2.6
*Aspergillus* spp*.*	1	0.1	-	-

*including *Providencia stuartii*, *Stenotrophomonas maltophilia*, *Aeromonas* spp., *Bacteroides fragilis*, and *Hafnia alvei*.

^#^*Hafnia alvei* as the pathogen*.*

From 187 complex sternal site infections (Table 3), 152 (81.3%) had pathogenic organisms isolated. Gram-positive bacteria (e.g. MSSA, MRSA and coagulase-negative *Staphylococci*) contributed to 70.4% of the infections, Gram-negative bacteria (e.g. *Serratia marcescens, Enterobacter* spp., *Klebsiella* spp., *Pseudomonas aeruginosa*) 27.0%, and *Candida albicans* 2.6%.

### Risk factors for complex sternal site infections

The univariate analysis (Table 4) identified five variables as potential risk factors: ASA score of 4/5, emergency surgery, CABG with sternal site incisions only, number of grafts, and absence of documentation of antibiotic prophylaxis. Two of these variables were retained in the multivariate logistic regression model as risk factors for complex sternal site infections (Table 5): ASA score of 4 or 5 (in relation to score of 3, OR 1.83, 95% CI 1.36-2.47) and absence of documentation of antibiotic prophylaxis (OR 2.03, 95% CI 1.12-3.69).

**Table 4:** Univariate analysis of risk factors for complex sternal site infections following coronary artery bypass graft procedures, Queensland public hospitals, 2003-2012

**Risk factor**	**Crude rate (per 100 procedures)**	**Odd ratio**	**95% CI**	***p***
Sex				
Female	1.2	Referent		
Male	1.3	1.05	0.73 – 1.49	0.796
Age category, years				
<55	1.2	Referent		
55-64	1.2	0.98	0.61 – 1.57	0.938
65-74	1.3	1.08	0.69 – 1.68	0.742
75+	1.5	1.29	0.81 – 2.07	0.286
ASA (American Society of Anaesthesiologists) score				
1/2	0.6	0.59	0.08 – 4.29	0.606
3	1.0	Referent		
4/5	1.7	1.73	1.29 – 2.32	<0.0001
Priority of surgery				
Elective	1.2	Referent		
Emergency	1.7	1.40	0.92 – 2.14	0.120
CABG types				
CABG with both sternal and harvest site incisions	1.2	Referent		
CABG with sternal site incisions only	2.3	1.87	1.17 – 2.99	0.009
Wound classification				
Clean	1.3	Referent		
Clean-contaminated	0.9	0.67	0.17 – 2.72	0.575
Number of grafts				
1	1.8	1.54	0.97 – 2.44	0.068
2	1.2	1.01	0.68 – 1.51	0.945
3	1.2	Referent		
4	1.5	1.30	0.89 – 1.88	0.171
5+	1.0	0.82	0.39 – 1.71	0.599
Antibiotic prophylaxis				
Yes	1.2	Referent		
No	2.6	2.09	1.15 – 3.78	0.015

**Table 5:** Multivariate analysis of risk factors for complex sternal site infections following coronary artery bypass graft procedures, Queensland public hospitals, 2003-2012

**Risk factor**	**Odd ratio**	**95% CI**	***p***	**Goodness-of-fit**	**C-index**
ASA (American Society of Anaesthesiologists) score				0.797	0.588*
3	Referent				
4/5	1.83	1.36 – 2.47	<0.0001		
Antibiotic prophylaxis					
Yes	Referent				
No	2.03	1.12 – 3.69	0.020		

*Validation of the model based on 100 bootstrap samples achieved a mean c-index of 0.579 (95% CI: 0.576 – 0.583), indicating good internal validation.

### Trend in rates of complex sternal site infections over time

**Figure 2:**
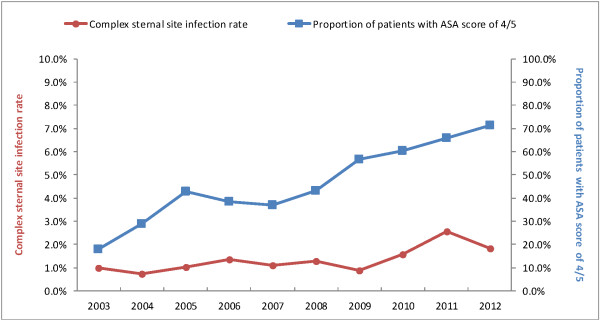
Trends in complex sternal site infection rates following coronary artery bypass graft procedures, and proportions of patients with ASA (American Society of Anaesthesiologists) score of 4/5, 2003–2012.

An overall upward trend in crude rates of complex sternal site infections was observed over the reporting period, ranging from 0.7% in 2004 to 2.6% in 2011 (Figure 2). There was a substantial increase in the proportion of CABG patients with ASA score of 4/5 (from 18.1% in 2003 to 71.3% in 2012, Figure 2). Proportions of patients with absence of documentation of antibiotic prophylaxis fluctuated between 1.8% and 4.8% over the reporting period.

## Discussion

The complex sternal site infection rate (1.3%) in our study is comparable with findings from the HAI surveillance system in Norway (1.1%, 2,440 CABG procedures) [19] and the NHSN system in the US (1.2%, 133,503 procedures) [14]. Characteristics of patients and CABG procedures were similar across these three study settings in terms of compositions of gender, age and wound classification, and proportions of emergency procedures. However, a higher proportion of patients with ASA score of 4/5 (73%) was reported by the NHSN system, as compared to those from the Norwegian (27%) and our data (45%), indicating a higher severity of underlying disease among patients undergoing CABG surgery captured in the NHSN system. Furthermore, the complex sternal site infections reported by the NHSN excluded cases detected during post-discharge surveillance (cases detected at readmission were included), due to concern of inconsistency in post-discharge case finding across 293 participating hospitals. In contrast, both in-hospital and post-discharge detected infection cases were included in Norwegian and our data, where post-discharge SSI surveillance data were collected from a relatively small number of participating hospitals (six and three respectively).

Our data indicate the importance of Gram-negative bacteria as causative agents for SSIs following CABG procedures. As observed in three participating hospitals, proportions of all SSIs (superficial and complex at both the sternal and harvest sites) caused by Gram-negative organisms increased from 38% in 2003 to 62% in 2009, despite a reduction to 42% in 2012. Harrington and colleagues [6] reported that Gram-negative bacilli were isolated from 18% of SSIs after CABG surgery, based on surveillance data collected during 1998–2001 in Victoria, Australia, while the corresponding figure based on our surveillance data over 2003–2012 was 44%. Further analysis of pathogens causing complex sternal site infections in our study showed Gram-negative bacteria were responsible for 27% of these serious infections, which is consistent with recently published NHSN data (Gram-negative organisms accounting for 34% of pathogens in complex SSIs after CABG surgery) [20]. An earlier study conducted in the US during 2000–2004 found 11% of sternal SSIs were caused by Gram-negative aerobes [21]. The apparently increasing proportion of CABG surgical site infections caused by Gram-negative organisms has important implications for clinical practice. Widely used guidelines (for example, the Sanford Guide to Antimicrobial Therapy) recommend cefazolin, cefuroxime or vancomycin as perioperative prophylaxis for cardiovascular surgery [22]. The current Australian Therapeutic Guidelines recommend three options for antibiotic prophylaxis in cardiac surgery: cefazolin alone, a combination of flucloxacillin and gentamicin, or a combination of vancomycin and gentamicin [23]. *Psedomonas aeruginosa* is intrinsically resistant to first generation cephalosporins. Other major Gram-negative organisms reported in our study (e.g. *Enterobacter* spp. and *Serratia marcescens*) belong to a genus whose inducible beta-lactamase production is a common property [9]. Given the increased prevalence of Gram-negative organisms associated with SSIs, we believe that cardiac surgeons should consult with their microbiologists to discuss local antibiograms prior to selecting surgical prophylactic antibiotics for patients undergoing CABG procedures.

Patient-based active surveillance data on CABG procedures provide an opportunity to investigate the long term trend in SSI rates. An increase in complex sternal site infection rates over 2003–2012 can be partially explained by variation in case mix of patients. There was a substantial increase in the proportion of patients with ASA score of 4/5 (indicating severe underlying disease) from 18% to 71% over the last decade. This might reflect the changing landscape of coronary intervention characterised by more patients with coronary artery disease undergoing treatment by percutaneous revascularisation (e.g. balloon angioplasty and stenting), while surgical revascularisation (CABG) is reserved for patients with more comorbid conditions and more severe underlying disease [24]. This study provides evidence on the increased risk of surgical site infections driven by increasing severity of illness among the patient population electing for CABG surgery.

The relatively poor discriminatory ability of the NNIS risk index for CABG surgical site infections has been widely reported across various study settings [11,14,19,25-27]. The underlying reason is that nearly all patients undergoing CABG surgery would have an ASA score ≥ 3 and have wound classification of clean or clean-contaminated, leaving the NNIS risk index differentiating patients just based on the procedure duration (top quartile vs. the rest). In this study, we re-categorised the ASA score into three groups (1/2, 3, and 4/5) to differentiate host susceptibility to infection for epidemiological purposes. Use of alternative risk scores (such as the EuroSCORE [26] and the Admission-specific Chronic Disease Scores [25]) has been associated with improved predictive performance for SSIs following CABG procedures. However, construction of these risk scores requires extensive and complex clinical data; their application to routine SSI surveillance data is subject to advancements of the underlying surveillance systems.

There is increasing interest in developing risk adjustment models for appropriate comparison of SSI rates following CABG surgery as part of publicly available hospital performance metrics. A recently published risk adjustment model based on the NHSN surveillance data included five predictors (ASA score, procedure duration, medical school affiliation, and interaction of age and sex) [14], achieving predictive performance of 0.62 (c-index) for complex sternal site infections after CABG surgery. A similar study (from Victoria, Australia) reported a predictive performance of 0.64 (c-index) for a model containing two predictors: diabetes, and body mass index (BMI > 35) [27]. In our study, a logistic regression model with two independent predictors (ASA score; absence of documentation of antibiotic prophylaxis) reported a c-index of 0.59 in predicting complex sternal site infections following CABG surgery. It appears that inclusion of a common set of patient/procedural factors such as ASA score, procedure duration, diabetes status, obesity (BMI) and antibiotic prophylaxis status in a risk adjustment model would enhance our understanding of the extent to which these factors contribute to and predict the SSIs after CABG surgery. However, none of the models mentioned above was able to test this full set of potentially important predictors, due to lack of data on one or more factors in their respective HAI surveillance systems. As not reaching a c-index of 0.7, considered to be indicative of acceptable predictive performance, those risk adjustment models may not be sufficient for appropriate comparison of infection rates across hospitals.

There were some limitations associated with our HAI surveillance data. First, the standardised surveillance data on CABG surgical site infections were collected from a total of three public hospitals which had capacity to provide CABG surgery for all public patients in Queensland. However, patients undergoing CABG at private hospitals (accounting for approximately 40% of the total patients) [28] were excluded from surveillance, thus limiting our ability to generalise findings to the patient population in Queensland as a whole. Second, there was concern regarding inconsistency in detecting SSIs (particularly the superficial infections) at post-discharge surveillance, due to inter-hospital variation in terms of completeness of follow-ups and potentially unreliable diagnosis of infections based on patient self-reporting [29]. This can be partly addressed by focusing on complex sternal site infections, which are more likely to be reported by patients at post-discharge surveillance and diagnosed by clinicians. Our data indicated 41% of complex sternal site infections were detected following discharge. In addition, complex sternal site infections have a greater consequence for patients. Third, procedure duration was not included as a potential risk factor for CABG surgical site infections due to concern with inconsistency in collecting this information, thus limiting our ability to assess its role in predicting surgical site infections.

With transition of the eICAT to a more sophisticated Multiprac^©^ surveillance system in Queensland Health, which features automatic linkages to multiple patient demographic and clinical databases, additional data on relevant risk factors (e.g. diabetes status, isolated CABG procedures or in conjunction with valve replacements) and infection outcomes will be routinely collected. There is scope to further refine our logistic regression model for risk adjustment, and to assist ongoing monitoring, public reporting, and improving surgical site infection control practice in hospitals.

## Conclusions

Our analysis of 10 years of CABG surgical site infection surveillance data indicates the importance of Gram-negative organisms as causative pathogens, and emphasises the need to select appropriate prophylactic antibiotics for patients undergoing CABG procedures. An upward trend in complex sternal site infection rates can be partially explained by the increasing proportion of CABG patients with more severe underlying disease. Future research should focus on development of appropriate and adequate risk adjustment models to facilitate valid comparison of CABG surgical site infection rates across hospitals.

## Competing interests

The authors declare that they have no competing interests.

## Authors’ contributions

DS contributed to study design, performed the statistical analysis, and drafted the manuscript. MR contributed to data management and analysis. JM, PL and MR contributed to study conceptualisation. JM supervised the data analysis and provided a major role in revising the manuscript. PL, CC and DP contributed to interpretation of results, editing and revising the manuscript. All authors contributed to, have read and approved the final manuscript.

## Pre-publication history

The pre-publication history for this paper can be accessed here:

http://www.biomedcentral.com/1471-2334/14/318/prepub
